# Perceptions of Romanian Physicians on Lockdowns for COVID-19 Prevention

**DOI:** 10.3390/healthcare9010095

**Published:** 2021-01-18

**Authors:** Alina Dima, Daniel Vasile Balaban, Ciprian Jurcut, Ioana Berza, Ruxandra Jurcut, Mariana Jinga

**Affiliations:** 1 Colentina Clinical Hospital, 072202 Bucharest, Romania; alina_dima@outlook.com (A.D.); ioana_berza@yahoo.com (I.B.); 2 Internal Medicine and Gastroenterology Clinic, Carol Davila University of Medicine and Pharmacy, 030167 Bucharest, Romania; rjurcut@gmail.com (R.J.); mariana_jinga@yahoo.com (M.J.); 3 Dr. Carol Davila Central University Emergency Military Hospital, 010825 Bucharest, Romania; cjurcut@gmail.com; 4 CC Iliescu Institute of Cardiovascular Diseases, 022322 Bucharest, Romania

**Keywords:** SARS-CoV-2, COVID-19, lockdown, physicians’ perception, survey, questionnaire

## Abstract

The severe acute respiratory syndrome coronavirus 2 (SARS-CoV-2) disease (COVID-19) was declared a pandemic in March 2020, triggering important changes for the entire society and healthcare systems, as well as significant lockdown measures aimed to limit the disease spread. We herein intended to catch the dynamic of Romanian physicians’ perceptions of COVID-19 impact. For this purpose, after a literature review, a 30-item questionnaire was designed. The questionnaire was twice distributed online, about 1 month apart, during which partial relaxation measures were decreed in Romania. The questionnaire was voluntarily filled in by Romanian physicians who were willing to participate in the study. A total of 214 physicians answered the questionnaire upon its first release, and 199 respondents were registered upon its second release, most of whom (94.9%) were involved in clinical work, with one-third working in units dedicated to COVID-19 patients. In parallel with the relaxation of lockdown measures, along with increased confidence in the efficiency of protective measures (46.7% vs. 31.3%), separation from household members decreased from 36.9% to 22.1%. Nevertheless, the feeling of rejection felt by doctors remained similar (22.4% vs. 24.6%). Furthermore, answers regarding the clinical picture, diagnostic approach, and treatment options are discussed. Most of therapeutic options considered for SARS-CoV-2 treatment (e.g., lopinavir/ritonavir, oseltamivir, hydroxychloroquine, azithromycin, tocilizumab, and convalescent plasma) failed to confirm significant efficiency. On the contrary, vaccines for widescale use are already available despite the initial skepticism. In the beginning of the pandemic, 25.2% (18.2% vs. 32.2%) considered that there will not be an effective COVID-19 vaccine, while 41.6% (43.0% vs. 40.2%) thought that a vaccine would be available after at least 12 months. In conclusion, initially, following only a 1 month period, Romanian physicians’ intention to consider treatments such as hydroxychloroquine or lopinavir/ritonavir for COVID-19 decreased significantly. Moreover, confidence in the efficiency of available protective measures increased, and the rates of separation from household members decreased.

## 1. Introduction

In late December 2019, a cluster of viral pneumonia cases of unknown origin was reported in Wuhan, China. The causative pathogen of the outbreak was reported to be a novel coronavirus, closely related to those responsible for previous outbreaks, i.e., severe acute respiratory syndrome coronavirus (SARS-CoV) and Middle East respiratory syndrome coronavirus (MERS). Coronaviruses typically cause respiratory, gastrointestinal, and neurological disease [1,2]. The new virus was identified as SARS-CoV-2, and the associated disease was named COVID-19 [1].

On 11 March 2020, the World Health Organization (WHO) declared COVID-19 a pandemic [3]. As of December 2020, over 70 million cases and more than 1.5 million deaths due to COVID-19 have been reported [4], despite worldwide efforts in fighting this disease.

From the beginning of the pandemic, many countries set lockdown measures to limit spread of the disease. Restrictions and lockdowns imposed by authorities have caused significant social, economic, and healthcare disruptions. Phased unlocking was then gradually conducted, which opened economic and social activities. Healthcare systems were most impacted by the challenges of an unknown disease, shortages of personal protective equipment, the need to reorganize infrastructure and human resources, and restrictions imposed on patients in medical units.

Physicians worldwide were doubly impacted by COVID-19, on a personal level and as frontline fighters with the rapidly spreading pandemic. Our aim was to assess Romanian physicians’ perspectives on COVID-19 and the dynamic of their perception of its main aspects, during and after the lockdown phase, in a rapidly changing landscape of social-distancing rules and continuously updating medical knowledge.

A 30-question survey addressing physicians’ beliefs on COVID-19 was conducted by the authors during the peak months of the pandemic, revealing several gaps and uncertainties regarding COVID-19 knowledge worldwide [5].

## 2. Methods

### 2.1. Survey Design and Study Participants

The study presented here is an online questionnaire-based survey related to the main COVID-19 features. After a literature review, a set of 30 questions was designed to investigate Romanian physicians’ perceptions regarding both personal impact and main COVID-19 epidemiological, diagnostic, and treatment features (see File S1, Supplementary Materials) [5].

The survey was introduced on a dedicated platform for online questionnaires and distributed twice, each time for 10 days. The first round of the survey was initially conducted during the national lockdown in Romania, in the so-called State of Emergency [6,7], and it opened on 24 April 2020. The same survey was reopened in a second round after partial unlocking, i.e., after the decree of the State of Alert [8], and it was re-distributed online on 25 May 2020. Each time, the questionnaire was distributed by the study authors via social networks. The survey was shared via social networks (Facebook, Twitter, LinkedIn) by the article’s authors and distributed especially in large online groups of Romanian physicians.

All Romanian physicians included voluntarily decided to take part in the survey and completed it by accessing the survey link. The questionnaire page had an introductory section that presented the questionnaire’s general structure, as well as the purpose and final goals of publishing the results obtained (see File S1, Supplementary Materials). Furthermore, the first items of the survey were designed as filter questions to select only Romanian physicians as respondents to the questionnaire.

The same 30-question survey addressing the demographic profile, COVID-19 features, and the personal impact on respondents was reopened for Romanian physicians after the lockdown phase. The second survey was generated using the same platform and also distributed online through social media. A sample size of 382 measurements was calculated for a 95% confidence interval (CI) and a 5% margin of error.

Only responses collected from physicians practicing in Romania were selected for the current research. A comparison of physicians’ answers during lockdown (initial questionnaire, entitled the first questionnaire) vs. the post-lockdown phase (reopened questionnaire, entitled the second questionnaire) was performed.

### 2.2. Ethics Approval

The participation in this study by completing the abovementioned questionnaire was completely voluntary and implied no risk for the respondents. Filling out the questionnaire was done after reading the information page on the survey and implied the informed consent for the inclusion in study. The Ethics Committee of the University of Medicine and Pharmacy approved this research.

### 2.3. Statistical Analysis

The statistical approach was descriptive, and categorical variables were expressed as numbers and percentages. Moreover, chi-square and Mann-Whitney tests were used to compare the responses given to the questionnaire before and after the change in lockdown measures. Furthermore, a comparison of proportions for the chi-square test was added. A *p*-value less than 0.05 was considered statistically significant. The SPSS V25 Software (IBM Corp, Armonk, NY, USA) and MedCalc 19.6.1 Software Ltd. (MedCalc, Ostend, Belgium) was used.

## 3. Results

### 3.1. Demographic Profile of Respondents

Altogether, 214 physicians during the first lockdown and 199 physicians after the start of the unlocking phase with less severe restriction measures completed the questionnaire. Most of the respondents (94.9%) were involved in clinical work during the pandemic. With regard to the medical specialty of the physicians included, there were 2 physicians in allergology (0 and 2), 6 in anesthesiology (3 and 3), 2 in cardiac surgery (2 and 0), 54 cardiology (30 and 24), 8 in dentistry (4 and 4), 11 in dermatology (3 and 8), 9 in diabetes (7 and 2), 14 in emergency medicine (2 and 12), 8 in endocrinology (5 and 3), 8 in ENT (6 and 2), 2 in epidemiology (none and 2), 19 in family medicine (6 and 13), 24 in gastroenterology (18 and 6), 6 in general surgery (3 and 3), 1 in geriatrics (none and 1), 1 in hematology (none and 1), 11 in infectious diseases (4 and 7), 7 in intensive care (6 and 1), 46 in internal medicine (29 and 17), 5 in laboratory medicine (1 and 4), 5 in neonatology (1 and 4), 6 in nephrology (1 and 5), 11 in neurology (7 and 4), 1 in neuropediatrics (0 and 1), 3 in neurosurgery (3 and 0), 9 in gynecology (7 and 2), 2 in occupational medicine (0 and 2), 4 in oncology (3 and 1), 12 in ophthalmology (8 and 4), 5 in orthopedics (3 and 2), 13 in other (10 and 3), 2 in pathology (0 and 2), 11 in pediatrics (1 and 11), 2 in physical medicine (0 and 2), 9 in pneumology (2 and 7), 1 in preventive medicine (0 and 1), 8 in psychiatry (2 and 6), 19 in radiology (8 and 11), 1 in radiation oncology (0 and 1), 45 in rheumatology (27 and 18), and 2 in urology (1 and 1) (see File S2, Supplementary Materials).

With regard to the age distribution of the survey participants, there were more seniors over 50 years of age among the respondents of the second questionnaire (17.6% vs. 0.4%), which was paralleled by a higher proportion of more experienced specialists (44.7% vs. 36%). There was an imbalance regarding the gender of survey respondents, with more males having completed the first questionnaire (29.4% vs. 17.6%)—Table 1.

### 3.2. Impact of COVID-19 on a Personal Level

Little over one-fourth of respondents in the first questionnaire and over one-third in the second one were working in a clinical unit dedicated to or partly reorganized for patients with COVID-19. Very few participants from both time periods had previously contracted COVID-19 (2.4% and 2% respectively). Possible contact with COVID-19 and feelings of rejections were reported in a similar proportion by respondents in the two time frames. Statistically significantly fewer physicians were separated from their household members after the lift of lockdown (22.1% vs. 36.9%). Moreover, an increase in trusting the efficacy of protective measures was reported after the lockdown phase (46.7% vs. 31.3%). Nevertheless, three in four physicians still considered that COVID-19-free patients continued to be neglected even after unlocking began in Romania—Table 2.

### 3.3. Diagnosis and Clinical Features of Covid-19

Awareness of COVID-19-presenting symptoms remained mostly unchanged between the two time frames, except for a lower proportion of respondents considering cough, abdominal pain, and cutaneous eruptions as important clinical manifestations of the disease. Regarding the diagnostic approach, nasopharyngeal reverse-transcriptase polymerase chain reaction (RT-PCR) SARS-CoV-2 was considered the first-line test in diagnosing SARS-CoV-2 infection by three in four physicians in both questionnaires. In the re-opened version of the questionnaire, more doctors considered chest computed tomography (CT) an important diagnostic test in COVID-19 patients (18.1% vs. 11.7%). Concerning disease prognosis, C-reactive protein, lymphocyte counts, and D-dimer levels were considered the three most important laboratory parameters with prognostic value by respondents of both questionnaires, while cardiac markers such as troponin and N-terminal pro b-type natriuretic peptide (NT-proBNP) were less taken into consideration by doctors in the second survey (19.6% vs. 28% for troponin; 15.6% vs. 24.3% for NT-proBNP)—Table 3.

There was wide consensus regarding the respiratory transmission pathway of SARS-CoV-2 among participants from both surveys, while, in the second questionnaire, fewer respondents were convinced about alternative infectious routes such as fecal–oral transmission (32.2% vs. 40.2%) or contact with contaminated objects (75.9% vs. 84.1%)—Table 4.

### 3.4. Treatment Options and Prognosis in COVID-19

Physicians’ beliefs about the impact of comorbidities on COVID-19 outcome did not significantly change during vs. post-lockdown phase, except for arterial hypertension and heart failure, which were less considered as having a negative prognosis on outcomes by respondents of the second questionnaire (64.3% vs. 72.4% for high blood pressure and 69.8% vs. 83.2% for heart failure)—Table 5.

There were significant differences among respondents regarding COVID-19 treatment; the most significant drop was seen in hydroxychloroquine and azithromycin, halved in respondents in the second questionnaire compared to the first one (26.6% vs. 51.9% for hydroxychloroquine and 23.1% vs. 40.7% for azithromycin). Others such as lopinavir/ritonavir, tocilizumab, remdesivir, and convalescent plasma were also less considered as curative by physicians in the last part of May when compared to only 1 month earlier.

With respect to the negative impact of preexisting medication, a lower proportion of respondents in the second survey mentioned nonsteroidal anti-inflammatory drugs (NSAIDs), angiotensin-converting enzyme (ACE) inhibitors, and angiotensin II receptor blockers (ARBs), but immunosuppressive drugs were still considered at-risk preexisting medication by half of physicians in both time periods—Table 6.

### 3.5. Perspectives on COVID-19 Prevention and Knowledge

Skepticism around an effective antiviral treatment and vaccine significantly increased among respondents in the post-lockdown phase compared to the beginning of the pandemic—Table 7. Doctors taking part in the second survey were still getting the information on COVID-19 mostly from medical journals (87.4%) or websites of scientific societies (79.9%), relying less on hospital protocols and social media. However, even fewer physicians were satisfied with the information they were getting on COVID-19 (37.2% vs. 44.9%)—Table 8.

## 4. Discussion

### 4.1. Epidemiology of SARS-CoV-2 Infection

In the beginning of 2020, COVID-19 unprecedentedly struck the healthcare systems as a novel, rapidly spreading, and unknown threat. Worldwide, huge efforts have been made in the last few months to increase the knowledge on COVID-19 and search for efficacious treatments and vaccines. Initially being thought of as a respiratory disease, COVID-19 is now known as a complex disease that can present with different clinical phenotypes [9] and the disease pathogenesis has multiple determinants [10].

The questionnaire we designed was distributed in the first months of the SARS-CoV-2 pandemic. Even so, 6 months later, knowledge on COVID-19, especially regarding a curative treatment, remains limited.

Along with the many gaps in knowledge about COVID-19, the pandemic has put tremendous pressure on healthcare systems, which had to undergo quick reorganizing of resources and infrastructure. Unlike other European Union countries, the medical units in Romania were divided into hospitals dedicated to COVID-19 patients vs. COVID-19-free hospitals, from where patients who tested SARS-CoV-2-positive were transferred to the care of the first units. Therefore, about one-third of the study respondents affirmed a work setting for COVID-19 patients.

With regard to the dynamic of the COVID-19 pandemic in Romania, it is important to note that more than five million Romanians are abroad and that, in March 2020, more than 250,000 Romanians returned in Romania [11]. Additionally, an important part of the Romanian diaspora is concentrated in the North of Italy [12]. Therefore, severe measures of social distancing during the State of Emergency (15 March–15 May 2020) were initially implemented that delayed the overburdening of the Romanian healthcare system that already had issues [5,6,7,11].

In Romania, the first case of SARS-CoV-2 infection was confirmed on 26 February 2020, a contact of an Italian citizen [13], and, 1 month later, on 22 March 2020, the first COVID-19 death was noted [14]. Among the first 147 COVID-19 cases in Romania, 88 were imported cases, with 64 from Italy [12]. At the first release of the present survey, there were around 9000 cases, more than 40,000 people quarantined at home, and more than 500 deaths from COVID-19 [14]. Even if many distancing rules were maintained for almost the entire year, including schools closing [15], the social pressure made it impossible and unreasonable to reimpose strict measures as in the beginning of the pandemic. Therefore, moments of overload for the Romanian healthcare system along with exceeding the capacity of Intensive Care Unit beds were noted during the pandemic. The risk of burnout for the medical staff was also increased, similarly to other regions [16]. In the beginning of 2021, the COVID-19 record in Romania is more than 600,000 cases and almost 19,000 deaths [17].

COVID-19 impacted healthcare workers worldwide, and Romanian physicians were also affected, recognizing an increased level of stress [18]. The answers to our questionnaire showed that COVID-19 patients were neglected, and the lower number of non-COVID cases treated might have a further impact on the training of young Romanian physicians [18].

The main transmission pathway involves droplets expelled in face-to-face exposure during talking, coughing, or sneezing, especially in conditions of longer than 15 min exposure and less than 2 m distance [2,19]. Transmission via aerosols (smaller particles) is also presumed. Moreover, RNA SARS-CoV-2 might be detected in blood and stool specimens [19]. Furthermore, it was reported that the virus might persist for 3–4 days on impermeable surfaces such as stainless steel and plastic [2]. Although theoretically possible, fomite transmission is difficult to prove because individuals who meet contaminated surfaces usually also have close contact with the infectious patient. Respondents in our survey readily recognized the respiratory route as the main transmission pathway of SARS-CoV-2, but also considered contact with contaminated objects a possible infectious route in high proportion.

From the infective contact, median incubation time is 2–12 days (median 5 days) [1]. After symptom occurrence, infected individuals are contagious for approximately 8 days [1], although some have reported that patients are most probably contagious a few days before the onset of COVID-19 symptoms [20].

During the time frame between the two releases of the questionnaire, the proportion of physicians working in a clinical setting, who assumed possible contact with COVID-19 patients, having feelings of rejection, or having COVID-19 did not change significantly, while the separation of the household members decreased in parallel with the increase in trust in the efficiency of protective measures.

The sources of information about SARS-CoV-2 infection accessed by Romanian physicians remained similar during and after lockdown, with medical journals, scientific societies websites, and internal hospital protocols being most used. Moreover, about 40% of the respondents obtained their information from social media channels. Unlike most published studies on a similar theme, one study showed that, for Romanian people, the exposure to COVID-19 information through social media was not related to self-assessed anxiety or depression [21].

### 4.2. Pathogenesis and Clinical Features of SARS-CoV-2 Infection

The SARS-CoV-2 targets the nasal and bronchial epithelial cells and pneumocytes using the viral structural spike (S) protein that binds the angiotensin-converting enzyme 2 (ACE2) receptor. Furthermore, in late stages, when the epithelial–endothelial barrier is affected, SARS-CoV-2 infects also the pulmonary endothelial cells [2].

The pulmonary phase of COVID-19 is driven by important immune dysregulation, endothelial damage, abnormal macrophage activation, vasculopathy centered by pulmonary microvascular impairment, and a procoagulant state with clotting activation that contributes to organizing pneumonia [22]. The use of corticosteroids in the early phase of the pulmonary stage is the only factor identified for improving outcomes [22]. Interstitial mononuclear inflammatory infiltrates and edema determine ground-glass pulmonary opacities, followed by hyaline membrane formation [2]. The pulmonary phase might further progress to an irreversible pulmonary fibroproliferative phase that is a distinct part of secondary organizing pneumonia and not necessarily acute respiratory distress syndrome (ARDS), at least initially [22]. As a difference, the ground glass infiltrates are mainly peripheral and patchy, unlike in ARDS [22]. In the final, fulminant phases, coagulation deficiency, microthrombus formation, and consumption of clotting factors occur [2].

The clinical symptomatology generally starts within the first days after contagious contact (in most cases, 5–11 days) [1]. About 40–45% of SARS-CoV-2-infected patients remain asymptomatic, with this pool of “silent spreaders” making control of the pandemic more difficult [23]. In particular, the proportion of asymptomatic persons was 58% on the cruise ship Diamond Princess at the time of testing; however, symptom onset might occur a few days after RT-PCR testing [24]. Others reported asymptomatic cases in only 4% (22,007 patients) of 616,541 confirmed cases [25].

Due to the broad expression of the ACE2 receptor throughout the human body (respiratory and gastrointestinal tract, endothelium) and complex pathogenesis of COVID-19, patients might present with a wide range of disease phenotypes, from flu-like symptoms, common for many viral respiratory infections (such as fever, headache, cough, and malaise), to digestive presentations, conjunctivitis, or Kawasaki-like multisystem inflammatory syndrome. Among 373,883 cases with data on individual symptoms expressed, 70% reported fever, cough, or shortness of breath, 36% reported muscle aches, and 34% reported headache; overall, 31,191 (8%) persons presented loss of smell or taste [25]. The clinical picture is centered by pneumonia occurrence in COVID-19, which manifests with bilateral, mainly peripheric pulmonary infiltrates, as well as fever, cough, and subsequent dyspnea [26]. Lai C-C et al. reviewed the extra-respiratory manifestations of COVID-19 and found cardiac (acute cardiac injury 8–12%, heart failure 23–52%, arrhythmia 8.9–16.7%, shock, acute myocarditis, chest tightness), gastrointestinal (anorexia 26.8%, diarrhea 12.5%, nausea and vomiting 10.2%, abdominal pain 9.2%), hepatic (increased aminotransferase levels 16.1–53.1%), renal (acute kidney injury 0.5%, 2.9–23% in severe cases), neurological (dizziness 16.8%, headache 13.1%, skeletal muscle injury 10.7%, impaired consciousness 7.5%, acute cerebrovascular disease 2.8%, ataxia 0.5%, seizures 0.5%), olfactory and gustatory (hyposmia 5.1–20.4%, anosmia 79.6%, dysgeusia 8.5%, ageusia 1.7%), ocular (acute conjunctivitis 31.6%), cutaneous (erythematous rash 15.9%, hives rash 3.4%, vesicles 1.1%), and hematological involvement (lymphopenia 56.5%, thrombocytopenia 16.4–32.3%, coagulation disorders, thrombotic events, antiphospholipid antibodies) [27]. In our survey, respondents in both time periods recognized the respiratory symptoms as the main clinical features of SARS-CoV-2 infection, but there was also a high response rate for extra-respiratory manifestations. Awareness of COVID-19-presenting symptoms remained mostly unchanged across the two time frames, except for a lower proportion of respondents considering cough, abdominal pain, and cutaneous eruptions as characteristic clinical manifestations of the disease. Similar data for symptomatology were presented in Romanian studies related to COVID-19 [11,14,28].

Some of the main adverse effects associated with the antiviral drugs used in COVID-19 might be confused and need differential diagnosis with the baseline disease symptomatology, such as nausea, abnormal hepatic function, skin rash, or kidney injury for remdesivir, diarrhea, nausea, abdominal pain, asthenia, headache, or abnormal hepatic function for lopinavir/ritonavir, nasopharyngitis, headache, transaminases elevation for tocilizumab, or ataxia, psychosis, and seizures for ivermectin [27].

With regard to severity of disease, a research describing the COVID-19 clinical spectrum on 44,415 patients identified mild cases in 81% (36,160 cases), severe cases in 14% (6168 cases), and critical disease in 5% (2087 cases) [29]. The overall case–fatality rate was 2.3% (1023 of 44,672 cases), with 14.8% in patients aged 80 years (208 of 1408 cases), while it was 49.0% in critical cases (1023 of 2087 cases) [29]. More than three in four patients hospitalized for COVID-19 patients need oxygen supplementation during admission [2]. Around 5% of all patients, as well as 20% of hospitalized COVID-19 patients, develop severe pathology with need for intensive care [2].

### 4.3. Diagnosis of SARS-CoV-2 Infection

Regarding the diagnostic approach, nasopharyngeal RT-PCR SARS-CoV-2 was considered the first-line test in diagnosing SARS-CoV-2 infection by three in four physicians in both questionnaires.

The specimens approved for the SARS-CoV-2 viral test are as follows: nasopharyngeal, oropharyngeal, nasal mid-turbinate swab, anterior nares (nasal swab), nasopharyngeal wash/aspirate, or nasal wash/aspirate specimen when collected by trained healthcare personnel or self-collected and observed by healthcare personnel, or by home or onsite self-collection (using a flocked or spun polyester swab), nasopharyngeal wash/aspirate or nasal wash/aspirate specimen collected by trained healthcare personnel, or a saliva specimen collected by the person being tested, either at home or at a testing site under supervision [30].

The RT-PCR from nasopharyngeal swab is the preferred and the main diagnostic test used for COVID-19. It is considered that the highest probability for a positive diagnostic RT-PCR nasopharyngeal is on day 8 of the SARS-CoV-2 infection. The probability of a false-negative result decreases from day 1 to day 4 from 100% to 67%. At the onset of symptoms, day 5, the median of false-negative tests is 38% (18–65%). The rate further decreases to 20–21% on day 8–9 and then returns to higher rates (66%) on day 21 [31].

After SARS-CoV-2 direct identification in culture, the virus can be identified for around 14 more days by RT-PCR SARS-CoV-2. After the viral replication phase, the RT-PCR SARS-CoV-2 test might remain positive, even though the patient is most probably no longer contagious.

Antigen testing is one alternative considered to regular methods involving nucleic acid amplification testing. One advantage is that antigen testing might be performed rapidly, leading to a faster time for obtaining results [32]. The average sensitivity for the rapid, point-of-care antigen and molecular-based tests for diagnosis of SARS-CoV-2 infection, according to 13 evaluations in 11 studies on 2255 samples, was 95.2% (95% CI 86.7–98.3%), with a specificity of 98.9% (95% CI 97.3–99.5%) [33].

The utility of serologic testing for COVID-19 diagnosis is considered limited now and not recommended for the first 14 days of infection [34]. This is due to lack of reactivity in the first days to weeks of infections and, therefore, the use of IgM SARS-CoV-2 for acute infection remains limited. In this context, it is especially recommended to test for the IgG serotype or for total antibody titers in persons with high probability of prior SARS-CoV-2 infection at about 3–4 weeks after the presumed infection [34].

Although chest CT was considered by a significant number of respondents as a meaningful diagnostic method for COVID-19 (14.8%), it cannot be used as first-line tool as patients might not have lung involvement in the beginning and it would be unreasonable in terms of time, cost, and radiation exposure [1]. Thorax CT scanning should, therefore, be considered later in the evolution of the disease, to appreciate the extent of lesions in moderate to severe patients and for risk stratification of those who might progress to lung failure and need intensive care. In one cohort of 201 hospitalized COVID-19 patients, 41.8% progressed to acute respiratory distress syndrome (ARDS), of which half died [35]; in this study, older age, hypertension, diabetes, neutrophilia, organ and coagulation dysfunction, and fever were associated with the risk of death [35]. Another study also found that age higher than 70 years, cardiovascular diseases, diabetes, corticosteroid use, body mass higher than 40 kg/m^2^, chronic kidney disease, and chronic obstructive pulmonary disease were underlying conditions associated with COVID-19 mortality [36]. Furthermore, pre-existent corticosteroid therapy was an important risk factor for COVID-19 severity [36]. The same study showed that current use of inhaled corticosteroids in chronic obstructive pulmonary disease was associated with a higher risk of severe disease [36]. The negative impact of comorbidities on COVID-19 outcome—i.e., heart failure, diabetes mellitus, arterial hypertension, chronic lung disease, neoplasia, chronic kidney disease, obesity [26,37]—was reported in high numbers by respondents in both questionnaires. Research has shown that comorbidities are also a risk factor for COVID-19 admission; if only about 25% of all COVID-19 patients have comorbidities, 60–90% of the hospitalized patients have associated comorbidities [2].

The laboratory parameters associated with severe COVID-19 outcome are higher than twofold D-dimer levels (more than 1000 ng/mL), C-reactive protein levels (more than 100 mg/L), lactate dehydrogenase levels (more than 245 units/L), more than twofold the normal upper limit for troponin, more than 500 mcg/L ferritin, and more than twofold the normal upper limit for creatinine-kinase [38]. Physicians in both questionnaires adequately recognized the prognostic value of the abovementioned markers, except for troponin, which was considered by only 24% of respondents as a negative prognostic marker.

### 4.4. Treatment of SARS-CoV-2 Infection

Different therapies were considered in COVID-19: antivirals (e.g., remdesivir, favipiravir), antibodies (e.g., convalescent plasma, hyperimmune immunoglobulins), anti-inflammatory agents (e.g., corticosteroids, statins), immunomodulatory therapies (e.g., tocilizumab, sarilumab, anakinra), anticoagulants (e.g., heparin), and antifibrotics (e.g., tyrosine kinase inhibitors) [2]. In March 2020, the Romanian Ministry of Health released a protocol for the treatment of SARS-CoV-2 infection [39], which was further updated in August 2020 [40]. The protocol released stated that there is no approved treatment for COVID-19 and that the current recommendation is based on ongoing international efforts. Furthermore, internal protocols for the COVID-19 units were developed in many Romanian hospitals.

Prophylaxis proposals for severe COVID-19 include zinc, quercetin, vitamin C, vitamin D, melatonin, famotidine, and ivermectin [22]. In vitro, zinc might inhibit the in vitro replication of SARS-CoV by interfering with RNA-dependent RNA polymerase [41]. Furthermore, zinc deficiency is related to increases in inflammatory responses [42]. The 3C-like protease has an important role in SARS-CoV-2 and was so identified as a possible target for antiviral therapies. Quercetin-3-beta-galactoside was identified as a natural inhibitor of the protease by molecular docking [43,44]. The benefits of quercetin might be also modulated by zinc actions; the ionophore actions of quercetin increase the levels of zinc [45]. The administration of quercetin and vitamin C might have synergic antiviral properties, as ascorbate might recycle quercetin increasing its efficacy [46]. Vitamin D insufficiency is also associated with some of the factors identified for severe prognosis in COVID-19, namely, hypertension, obesity, male sex, advanced age, coagulopathy, and immune dysfunction [47]. Vitamin D deficiency was found to be correlated with mortality in COVID-19 patients [48]. Ivermectin is proposed as treatment for the initial phase with viral replication, as well as during the pulmonary phase [22]. Melatonin is an endogenously produced molecule in small amounts with presumed anti-inflammatory, antioxidant, and antiviral effects [49]. Melatonin was considered as treatment in sepsis due to its anti-inflammatory, antiapoptotic, and powerful antioxidant properties [50] and further proposed for SARS-CoV-2 treatment [51].

Currently available treatments have different targets according to the infection stage. Therapies with antiviral effects are considered for the first phase of the viral disease to prevent and stop SARS-CoV-2 replication. Furthermore, for the infection pulmonary phase, interest is focused the anti-inflammatory therapies, particularly corticoids [52]. Because of the lack of effective therapies, treatment strategies are focused on the supportive management of acute hypoxic respiratory failure, related complications, and comorbidity decompensation [2].

With regard to antivirals, in the beginning of the pandemic there was high interest toward the second-generation antiretroviral drug combination lopinavir/ritonavir, which inhibits the SARS-CoV-2 viral protease [1]; however, studies failed to confirm its efficiency. Hydroxychloroquine was also considered in COVID-19 treatment because of previous research on SARS-CoV infection in 2002, as well as results regarding its in vitro inhibitory effects on SARS-CoV-2 replication [53,54]. However, hydroxychloroquine did not show significant benefits to standard care in COVID-19 [55] according to research including five randomized control trials [56]; moreover, hydroxychloroquine-related side effects were considered [57]. The hydroxychloroquine recommendation for COVID-19 treatment was, therefore, stopped [58]. Some guidelines are now recommending that ivermectin might have both antiviral and anti-inflammatory benefits [22]; however, more studies are still needed to confirm this hypothesis. To sum up, therapies with potential antiviral properties such as hydroxychloroquine, lopinavir-ritonavir, interferon α/β, and tocilizumab failed to be efficient in the COVID-19 symptomatic phase, and a trend to harm was also observed for hydroxychloroquine and interferon α/β when used in the late inflammatory disease stage [22].

High expectations were also set for antibody-based therapies; however, convalescent plasma and monoclonal antibodies are not considered for standard care in COVID-19, whereas their administration might be beneficial in early and very early infection stages [19]. One living systematic review states that the only therapeutic interventions with potential benefits in COVID-19 that remain are remdesivir and the glucocorticoids, with corticosteroids being the only therapy with an impact on mortality [55]. In our survey, respondents in both time periods reported high confidence with the use of plasma from convalescent donors as a treatment for COVID-19. Furthermore, about one in two respondents considered remdesivir as curative for SARS-CoV-2 infection. Overall, the response dynamics followed the decrease in evidence for the therapeutics considered.

Remdesivir is an inhibitor of RNA-dependent RNA polymerase [19] that was used as an antiviral in SARS-CoV and MERS infections by impairing the viral polymerase of coronavirus [1]. Remdesivir is recommended in COVID-19 patients with severe disease, while data are insufficient for use in less severe disease [19]. Five days of use seems to have similar results to 10 days of use [19].

There is a trend to harm for corticosteroid use when administrated in the symptomatic phase, but significant benefits are associated with use during inflammatory stages [22]. Corticosteroids such as methylprednisolone decrease the risk of progression to ARDS [35]. In the RECOVERY study, 2104 COVID-19 patients were randomly administered 6 mg of dexamethasone daily for up to 10 days, and 4321 patients presented a reduction in all-cause mortality on day 28 [59]. Some guidelines recommend methylprednisolone use for the pulmonary phase of COVID-19 due to better penetration in the lung tissues, genomic data, and known results in inflammatory lung diseases [22]. An early short course of methylprednisolone (0.5 to 1 mg/kg/day divided into two intravenous doses for 3 days) in patients with moderate to severe COVID-19 might reduce the escalation of care and improved clinical outcomes [60]. One meta-analysis regarding the association between systemic corticosteroid use and mortality among critically ill COVID-19 patients showed lower 28 day all-cause mortality for patients under systemic corticosteroids, compared with usual care or placebo [61].

Broad-spectrum empirical antimicrobials are frequently prescribed in COVID-19, but there is a paucity of data to support this approach [62]. For COVID-19, even if only about 8% patients presented bacterial or fungal coinfection during hospital admission, broad-spectrum antibacterial was used in 72% of cases [2], and this approach raises great concerns.

Anticoagulation, generally by low-molecular-weight heparin (WMWH), is presumed to act synergistically with corticosteroids and vitamin C for the endothelium protection [22]. About two-thirds of physicians who took part in the survey recognized the beneficial effect of anticoagulation in COVID-19. The doses and duration of thromboprophylaxis are recommended in hospitalized patients with COVID-19 according to the associated risk factors such as advanced age, hospitalization length, concurrent pro-thrombotic condition, severe immobilization, elevated D-dimers (more than twofold the normal upper limit, higher than 1000 ng/mL) [63]. The platelet count and the renal clearance level should also be monitored when deciding the anticoagulation doses [63]. Anti-Xa level monitoring should be considered in patients with low or high weight (<50 kg; >150 kg), impaired creatinine clearance, bleeding, or new thrombosis in order to avoid nontoxic levels and to assure therapeutic levels [63]. Both coagulopathy and thrombocytopenia might be signs of disseminated intravascular coagulopathy (CID) in COVID-19 patients, and parameters such as platelet count, D-dimer, fibrinogen, and prothrombin time need to be monitored [63].

In Romania, one study evaluating the outcome of hemodialysis patients reported that 60% received hydroxychloroquine, 22% received the lopinavir/ritonavir combination, 11% received tocilizumab, and 24% received systemic glucocorticoids, while only 54% received anticoagulation [64]. Even if azithromycin use was not recommended by the August 2020 protocol update of the Romanian Ministry of Health due to cardiovascular risk when administrated in parallel to hydroxychloroquine and the risk of antibiotherapy resistance in Romania, the use of hydroxychloroquine in COVID-19 was not contraindicated when no other alternative was available [40]; this was highly debated on Romanian physician forums. One interesting survey from the end of March 2020, which included 785 Romanian physicians, showed that even if 48% considered the evidence regarding hydroxychloroquine efficacy in COVID-19 acceptable and 91.5% recognized the need of a randomized controlled trial (RCT) on the subject, only 42% considering being enrolled in such a trial [65].

Regarding the negative impact of preexisting medication on COVID-19 outcome, respondents in our survey mostly considered NSAIDs (40.7%) and immunosuppressive drugs (53.5%). One retrospective cohort study that included 4480 patients with COVID-19 showed that prior use of ACE inhibitors or ARBs in patients with arterial hypertension was not associated with severe disease or mortality [66]. Moreover, there were not sufficient data to support the initial raised concerns related to NSAIDs in COVID-19. Acetaminophen remains the first-line symptomatic treatment in COVID-19 [19].

While no breakthrough treatment has so far been developed for COVID-19, significant progress has been made for prophylactic vaccines. Respondents in our survey were in line with this trend, with about one in four physicians considering that there will be no effective antiviral treatment but that a vaccine will be available. Several different platforms were developed for SARS-CoV2 vaccines, including traditional approaches such as inactivated viruses and live-attenuated viruses or newer methods such as recombinant proteins and vectors. There were also methods used for the first time, i.e., RNA and DNA vaccines [67]. At the end of 2020, there were 60 vaccines under development with 136 trials in 38 countries, 21 vaccines in phase 1, 24 vaccines in phase 2, and 15 vaccines in phase 3 of study [68]. There is no ongoing trial for a vaccine registered in Romania, even though there were attempts to develop an anti-SARS-CoV-2 vaccine.

The first vaccines approved in December 2020 for administration in the European Union countries are mRNA-based vaccines [67,69]. These vaccines are translated by the host’s cellular machinery [69]. Protection rates of 52% after the first dose and 95% after the second dose were reported [67]. However, the duration of protective immunity is not known. The minimum levels of anti-SARS-CoV-2 antibodies that confer protection is also currently unknown [19].

### 4.5. Study Limitations

A limitation of the current study is related to the continuous update in knowledge about COVID-19, which has dynamically changed practice several times since the beginning of the pandemic. However, it is also interesting to observe the physicians’ perspectives over time and compare the expectations from 6 months ago with the present reality. Our survey reflects the opinions of physicians in the first months of the pandemic, when evidence-based recommendations were lacking. Moreover, although the number of respondents was not as high as expected, we consider it representative given the age, professional degree, and distribution medical specialization.

## 5. Conclusions

In conclusion, our survey revealed a significant impact of COVID-19 for physicians on a personal level and in their medical practice. In a time frame of only 1 month, the physicians’ intention to prescribe treatments such as hydroxychloroquine and ritonavir/lopinavir combination decreased significantly. Furthermore, after 9 months of the ongoing pandemic, an efficient antiviral treatment is still lacking, and promising therapies in the early months proved disappointing. However, there was surprising progress made regarding vaccine prophylaxis for SARS-CoV-2 infection, overcoming the physicians’ expectations. Attention should be given to the fact that around three in four physicians considered that COVID-19 patients are neglected.

## Figures and Tables

**Table 1:** General data of the questionnaire’s responders.

Parameter	First *n*1 = 214	Second *n*2 = 199	*p*-Value
Clinical work, yes (%)	202 (94.4%)	190 (95.5%)	0.616
Age, *n* (%)			
20–29 years	52 (24.3%)	47 (23.6%)	0.136
30–39 years	92 (43.0%)	72 (36.2%)	
40–49 years	50 (23.4%)	45 (22.6%)	
50–59 years	16 (7.5%)	29 (14.6%)	
60–69 years	4 (1.9%)	5 (2.5%)	
70–79 years	0 (0.0%)	1 (0.5%)	
Gender, male (%)	63 (29.4%)	35 (17.6%)	0.005
Professional level, *n* (%)			
Resident/Intern/Fellow	62 (29.0%)	56 (28.1%)	0.253
Specialist <5 years of experience	66 (30.8%)	46 (23.1%)	
Specialist >5 years of experience	77 (36.0%)	89 (44.7)	
Head of Department/Professor	9 (4.2%)	8 (4.0)	

**Table 2:** Epidemiological data related to personal and work daily life of the questionnaire’s responders. COVID-19, severe acute respiratory syndrome coronavirus 2 (SARS-CoV-2) disease.

Parameter	First *n*1 = 214	Second *n*2 = 199	*p*-Value
Work setting for COVID-19 patients			
Yes	59 (27.6%)	69 (34.7%)	0.119
No	155 (72.4%)	130 (65.3%)	
Possible contact with COVID-19 patients			
Yes	169 (79.0%)	155 (77.9%)	0.897
No	11 (5.1%)	16 (8.0%)	
Not sure	34 (15.9%)	28 (14.1%)	
Feeling of rejection			
Yes	48 (22.4%)	49 (24.6%)	0.785
No	84 (39.3%)	74 (37.2%)	
Not applicable	82 (38.3%)	76 (38.2%)	
Personal COVID-19 diagnosis			
No	170 (79.4%)	165 (82.9%)	0.367
Yes, asymptomatic	4 (1.9%)	3 (1.5%)	
Yes, symptomatic	1 (0.5%)	1 (0.5%)	
Not sure	39 (18.2%)	30 (15.1%)	
Household COVID-19 diagnosis			
No	183 (85.5%)	171 (85.9%)	0.817
Yes, asymptomatic	1 (0.5%)	5 (2.5%)	
Yes, symptomatic	1 (0.5%)	2 (1.0%)	
Not sure	29 (13.6%)	21 (10.6%)	
Separation of household members			
Yes	79 (36.9%)	44 (22.1%)	0.001
No	130 (60.7%)	155 (77.9%)	
Efficiency of the protective measures			
Yes	67 (31.3%)	93 (46.7%)	0.040
No	72 (33.6%)	39 (19.6%)	
Not sure	75 (35.0%)	67 (33.7%)	
Neglect of COVID-19-free patients			
Yes	162 (75.7%)	150 (75.4%)	0.729
No	22 (10.3%)	25 (12.6%)	
Not sure	26 (12.1%)	24 (12.1%)	

**Table 3:** Clinical and paraclinical COVID-19 features.

Parameter	First *n*1 = 214	Second *n*2 = 199	*p*-Value
COVID-19 symptoms			
Fever	211 (98.6%)	192 (96.5%)	0.162
Cough	204 (95.3%)	177 (88.9%)	0.015
Dyspnea	204 (95.3%)	183 (92.0%)	0.159
Anosmia/ageusia	187 (87.4%)	167 (83.9%)	0.315
Abdominal pain	108 (50.5%)	83 (41.7%)	0.074
Diarrhea	154 (72.0%)	105 (52.8%)	0.000
Anorexia	44 (20.6%)	39 (19.6%)	0.807
Chest pain	92 (43.0%)	75 (37.7%)	0.273
Cutaneous eruptions	92 (43.0%)	68 (34.2%)	0.066
Conjunctivitis	52 (24.3%)	39 (19.6%)	0.249
Headache	129 (60.3%)	122 (61.3%)	0.831
Dysuria	3 (1.4%)	5 (2.5%)	0.413
Diagnostic approach			
Nasopharyngeal RT-PCR SARS-CoV-2	169 (79.0%)	151 (75.9%)	0.453
Stool RT-PCR SARS-CoV-2	2 (0.9%)	1 (0.5%)	0.606
IgM SARS-CoV-2	8 (3.7%)	8 (4.0%)	0.882
IgG SARS-CoV-2	6 (2.8%)	1 (0.5%)	0.071
Chest X-ray	1 (0.5%)	0 (0.0%)	0.335
Chest CT	25 (11.7%)	36 (18.1%)	0.067
Other	3 (1.4%)	2 (1.0%)	0.712
Prognostic marker			
Ferritin	121 (56.5%)	107 (53.8%)	0.571
C-reactive protein	170 (79.4%)	151 (75.9%)	0.385
Lymphocyte counts	160 (74.8%)	130 (65.3%)	0.036
Troponin	60 (28.0%)	39 (19.6%)	0.045
N-terminal pro b-type natriuretic peptide	52 (24.3%)	31 (15.6%)	0.027
D-dimers	136 (63.6%)	124 (62.3%)	0.794
SARS-CoV-2 viral load	122 (57.0%)	100 (50.3%)	0.169
None	1 (0.5%)	0 (0.0%)	0.334
Other	3 (1.4%)	13 (6.5%)	0.006

**Table 4:** Transmission pathways for SARS-CoV-2 virus.

Transmission Pathways	First *n*1 = 214	Second *n*2 = 199	*p*-Value
Respiratory	214 (100%)	198 (99.5%)	0.299
Fecal–oral route	86 (40.2%)	64 (32.2%)	0.090
Contact with contaminated objects	180 (84.1%)	151 (75.9%)	0.045
Others	2 (1.0%)	1 (0.5%)	-

**Table 5:** Impact of comorbidities on prognosis.

Parameter	First *n*1 = 214	Second *n*2 = 199	*p*-Value
Arterial hypertension	155 (72.4%)	128 (64.3%)	0.076
Heart failure	178 (83.2%)	139 (69.8%)	0.001
Chronic respiratory failure	192 (89.7%)	160 (80.4%)	0.008
Chronic kidney disease (without dialysis)	78 (36.4%)	66 (33.2%)	0.508
Chronic kidney disease (with dialysis)	185 (86.4%)	169 (84.9%)	0.658
Diabetes mellitus	205 (95.8%)	182 (91.5%)	0.070
Liver cirrhosis	110 (51.4%)	109 (54.8%)	0.493
Autoimmune pathology	106 (49.5%)	94 (47.2%)	0.641
Neoplasia	178 (83.2%)	157 (78.9%)	0.266
Obesity	175 (81.8%)	155 (77.9%)	0.325
No relation	0 (0.0%)	0 (0.0%)	-

**Table 6:** Therapy options in COVID-19. NSAIDs, nonsteroidal anti-inflammatory drugs; ACE, angiotensin-converting enzyme.

	First *n*1 = 214	Second *n*2 = 199	*p*-Value
Prevention			
Vitamin D	57 (26.6%)	58 (29.1%)	0.570
Zinc	40 (18.7%)	29 (14.6%)	0.262
Vitamin C	53 (24.8%)	50 (25.1%)	0.933
Hydroxychloroquine	45 (21.0%)	24 (12.1%)	0.015
Astragalus extract	4 (1.9%)	3 (1.5%)	0.776
Quercetin	8 (3.7%)	1 (0.5%)	0.024
*N*-Acetyl cysteine	6 (2.8%)	7 (3.5%)	0.678
None	119 (55.6%)	116 (58.3%)	0.582
Others	7 (3.3%)	10 (5.0%)	0.370
Curative treatment			
None	29 (13.6%)	50 (25.1%)	0.003
Paracetamol	97 (45.3%)	58 (29.1%)	0.001
Lopinavir/ritonavir	70 (32.7%)	42 (21.1%)	0.008
Oseltamivir	24 (11.2%)	15 (7.5%)	0.202
Hydroxychloroquine	111 (51.9%)	53 (26.6%)	0.000
Azithromycin	87 (40.7%)	46 (23.1%)	0.000
Tocilizumab	81 (37.9%)	60 (30.2%)	0.099
Remdesivir	109 (50.9%)	93 (46.7%)	0.393
Plasma from convalescent donors	145 (67.8%)	121 (60.8%)	0.140
Something else	4 (1.9%)	7 (3.5%)	0.298
Negative impact			
None	36 (16.8%)	47 (23.6%)	0.085
NSAIDs	98 (45.8%)	70 (35.2%)	0.028
ACE inhibitors	56 (26.2%)	27 (13.6%)	0.001
Sartans	20 (9.3%)	6 (3.0%)	0.008
Corticosteroids	64 (29.9%)	48 (24.1%)	0.186
Immunosuppressive drugs	109 (50.9%)	112 (56.3%)	0.276
Other (please specify)	4 (1.9%)	2 (1.0%)	0.460
Anticoagulation			0.065
Yes	132 (61.7%)	143 (71.9%)	
No	11 (5.1%)	6 (3.0%)	
Not sure	67 (31.3%)	50 (25.1%)	

**Table 7:** Perspectives on COVID-19 prevention options.

	First *n*1 = 214	Second *n*2 = 199	*p*-Value
COVID-19 effective vaccine			0.054
No	40 (18.7%)	64 (32.2%)	0.002
Yes, within the next 3 months	4 (1.9%)	2 (1.0%)	0.448
Yes, within the next 3–6 months	9 (4.2%)	8 (4.0%)	0.919
Yes, within the next 6–12 months	65 (30.4%)	45 (22.6%)	0.074
Yes, after at least 12 months	92 (43.0%)	80 (40.2%)	0.565
Effective antiviral treatment			0.124
No	36 (16.8%)	66 (33.2%)	<0.001
Yes, within the next 3 months	11 (5.1%)	8 (4.0%)	0.593
Yes, within the next 3–6 months	38 (17.8%)	20 (10.1%)	0.025
Yes, within the next 6–12 months	71 (33.2%)	47 (23.6%)	0.031
Yes, after at least 12 months	54 (25.2%)	58 (29.1%)	0.373
Reinfections			0.11
No	27 (12.6%)	61 (30.7%)	<0.001
Yes, in less than 6 months with the same viral strain	81 (37.9%)	42 (21.1%)	0.002
Yes, after at least 6 months with the same viral strain	23 (10.7%)	22 (11.1%)	0.896
Yes, after at least 12 months with the same viral strain	7 (3.3%)	4 (2.0%)	0.413
Yes, after at least 12 months with a new mutated viral strain	71 (33.2%)	70 (35.2%)	0.669
COVID-19 eradication			0.858
No, it will remain a permanent viral infection	128 (59.8%)	122 (61.3%)	0.756
Yes, within 3 months	3 (1.4%)	1 (0.5%)	0.351
Yes, within 6 months	4 (1.9%)	5 (2.5%)	0.678
Yes, within 12 months	14 (6.5%)	17 (8.5%)	0.44
Yes, after at least 24 months	61 (28.5%)	54 (27.1%)	0.751
COVID-19 periodic reactivation			0.678
Yes	49 (22.9%)	32 (16.1%)	0.082
No	47 (22.0%)	62 (31.2%)	0.034
Not sure	114 (53.3%)	105 (52.8%)	0.031

**Table 8:** Source of medical information and general data.

	First *n*1 = 214	Second *n*2 = 199	*p*-Value
Adequate information on COVID-19			
Yes	96 (44.9%)	74 (37.2%)	0.346
No	64 (29.9%)	83 (41.7%)	
Not sure	50 (23.4%)	42 (21.1%)	
Source of information on COVID-19			
Medical journals	187 (87.4%)	174 (87.4%)	0.987
Scientific societies websites	178 (83.2%)	159 (79.9%)	0.390
Internal hospital protocols at workplace	108 (50.5%)	89 (44.7%)	0.243
Hospital protocols from other than workplace	80 (37.4%)	83 (41.7%)	0.369
Social media	95 (44.4%)	71 (35.7%)	0.071
Other (please specify)	1 (0.5%)	5 (2.5%)	0.384

## Data Availability

Data used to support the findings of this study are included within the article. Any additional data are available from the corresponding author upon request.

## Supplementary Materials

The following are available online at https://www.mdpi.com/2227-9032/9/1/95/s1, Table S1: The questionnaire of the on-line survey; Table S2: Medical specialties of the survey respondents.
