# Supplementary material for: Perceptions of Romanian Physicians on Lockdowns for COVID-19 Prevention
Source: Healthcare (Basel). 2021 Jan 18;9(1):95. doi: 10.3390/healthcare9010095 (PMC7831077; doi:10.3390/healthcare9010095)
Supplement: Supplementary file 1 [file healthcare-09-00095-s001.pdf]

**Supplemental File 1**

## Physicians' perspectives on COVID-19

**Dear colleague,**
**Thank you for accepting our invitation to participate in this online survey dedicated to physicians worldwide, which evaluates current knowledge and experience during the COVID-19 pandemic. As we all know, the pressure on healthcare systems and the medical community are higher than ever before. Through this short online questionnaire, we invite practitioners all over the world to anonymously share their beliefs regarding COVID-19. The survey includes 30 single-choice or multiple-choice questions and takes around 5-6 minutes to complete it. Participating in this survey implies the agreement to use the answers in carrying out an analysis on the current knowledge and experience with COVID-19. Data gathered through this survey will be processed completely anonymously. By completing this questionnaire, you express your agreement to voluntarily participate in this question-based research. You can refuse to participate in the study or withdraw at any time before finishing the survey. Results will be presented in a research paper available online. For inquiries please contact physician.survey.covid19@gmail.com.**

**Questionnaire**

1. Are you a physician currently practicing medicine in a clinical setting?
   a. Yes
   b. No

2. What is your age?
   a. 20 – 29 years
   b. 30 – 39 years
   c. 40 – 49 years
   d. 50 – 59 years
   e. 60 – 69 years
   f. 70 – 79 years

3. What is your gender?
   a. Female
   b. Male

4. Are you currently practice medicine in Romania?
   a. Yes
   b. No

5. What is your medical specialty?
   Listed …

6. What is your current professional level?
   a. Resident physician/Intern/ Fellow
   b. Specialist physician with less than 5 years' experience
   c. Specialist physician with more than 5 years' experience
   d. Head of Department/ Professor

7. Do you work in a service/ department/ clinic dedicated to/or where patients with COVID-19 are hospitalized?
   a. Yes
   b. No

8. Do you believe you might come in contact with COVID-19 infected patients during consultations/medical procedures?
   a. Yes
   b. No
   c. Not sure

9. After starting to work in units where patients diagnosed with COVID-19 infection were hospitalized, did you feel avoided/ rejected by the persons with whom you usually interact in daily life?
   a. Yes
   b. No
   c. Not applicable

10. Have you already had COVID-19 yourself?
    a. No
    b. Yes, an asymptomatic form
    c. Yes, a symptomatic form
    d. Not sure

11. Has any of your household members had COVID-19?
    a. No
    b. Yes, an asymptomatic form
    c. Yes, a symptomatic form
    d. Not sure

12. Do you believe that the protective equipment, and clinic's procedures in triage and differentiated pathways, are enough to protect you?
    a. Yes
    b. No
    c. Not sure

13. Which of the following clinical symptoms do you think are suggestive for COVID-19? (multiple answers possible)

a. Fever
b. Cough
c. Dyspnea
d. Anosmia/ ageusia
e. Abdominal pain
f. Diarrhea
g. Anorexia
h. Chest pain
i. Cutaneous eruptions
j. Conjunctivitis
k. Headache
l. Dysuria

14. Which of the following tests has the highest diagnostic accuracy, in your opinion?
    a. RT-PCR SARS-CoV2 of the nasopharyngeal secretion
    b. RT-PCR SARS-CoV2 of stool sample
    c. RT-PCR SARS-CoV2 of the conjunctival secretion
    d. IgM SARS-CoV2
    e. IgG SARS-CoV2
    f. Chest X-ray
    g. Chest CT (computed tomography)
    h. Other

15. Which of the following associated comorbidities are negative prognostic factors for COVID-19, in your opinion?
    (multiple answers possible)
    a. Arterial hypertension
    b. Heart failure
    c. Chronic respiratory failure
    d. Chronic kidney disease (without dialysis)
    e. Chronic kidney disease (with dialysis)
    f. Diabetes mellitus
    g. Liver cirrhosis
    h. Autoimmune pathology
    i. Neoplasia
    j. Obesity
    k. COVID-19 disease course is not related to comorbidities

16. Which of the following blood test you think it is correlated to outcomes in COVID-19?
    (multiple answers possible)
    a. Ferritin
    b. C-reactive protein
    c. Lymphocyte counts
    d. Troponin

e. NT-proBNP
f. D-dimers
g. SARS-CoV2 viral load (RNAaemia)
h. Other

17. Which of the following do you think are potential transmission pathways for SARS-CoV-2 virus?
    (multiple answers possible)
    a. Respiratory
    b. Fecal-oral route
    c. Contact with contaminated objects
    d. Others

18. Do you think there is any useful therapy for the prevention of COVID-19?
    (multiple answers possible)
    a. Vitamin D
    b. Zinc
    c. Vitamin C
    d. Hydroxychloroquine
    e. Astragalus extract
    f. Quercetin
    g. N-acetyl Cysteine
    h. Other

19. Which of the following therapies do you think is effective in COVID-19?
    (multiple answers possible)
    a. No effective treatment exists
    b. Paracetamol
    c. Lopinavir/ Ritonavir
    d. Oseltamivir
    e. Hydroxychloroquine
    f. Azithromycin
    g. Tocilizumab
    h. Remdesivir
    i. Plasma from convalescent donors
    j. Something else

20. Is there any pre-existing medication that might worsen the prognosis of the COVID-19?
    (multiple answers possible)
    a. None
    b. NSAIDs
    c. ACE inhibitors
    d. Sartans

e. Corticosteroids
f. Immunosuppressive drugs
g. Other (please specify)

21. Do you think we will have an effective vaccine to prevent the COVID-19?
    a. No
    b. Yes, within the next 3 months
    c. Yes, within the next 3-6 months
    d. Yes, within the next 6-12 months
    e. Yes, after at least 12 months

22. Do you think we will have an effective antiviral treatment for COVID-19?
    a. No
    b. Yes, within the next 3 months
    c. Yes, within the next 3-6 months
    d. Yes, within the next 6-12 months
    e. Yes, after at least 12 months

23. Do you think there can be reinfections with COVID-19 can occur?
    a. No
    b. Yes, in less than 6 months with the same viral strain
    c. Yes, after at least 6 months with the same viral strain
    d. Yes, after at least 12 months with the same viral strain
    e. Yes, after at least 12 months with a new mutated viral strain

24. Do you think abnormal hemostasis plays a central role in the pathogenesis of COVID-19 and should be targeted by therapeutic anticoagulation?
    a. Yes
    b. No
    c. Not sure

25. Do you think it will be possible to eradicate COVID-19 infection?
    a. No, it will remain a permanent viral infection in the population
    b. Yes, within 3 months
    c. Yes, within 6 months
    d. Yes, within 12 months
    e. Yes, after at least 24 months

26. Dou you think it is possible to have persistence of SARS-CoV2 in the human body with subsequently periodic reactivation?
    a. Yes
    b. No
    c. Not sure

27. Do you consider that you receive enough information about COVID-19?
    a. Yes
    b. No
    c. Not sure

28. Where do you get medical/scientific information about COVID-19? (multiple answers possible)
    a. Medical journals
    b. Scientific societies websites
    c. Internal hospital protocols at workplace
    d. Hospital protocols from other than workplace
    e. Social media
    f. Other (please specify)

29. Do you think that COVID-19 free patients are medically neglected during this period?
    a. Yes
    b. No
    c. Not sure

30. Are you currently isolated from the people with whom you usually live with?
    a. Yes
    b. No

**Supplementary Table S2**. Medical specialties of the survey respondents

| Medical Specialty | First<br>*n*1 = 214 | Second<br>*n*2 = 199 |
|---|---|---|
| Allergology | - | 2 |
| Anesthesiology | 3 | 3 |
| Cardiac Surgery | 2 | - |
| Cardiology | 30 | 24 |
| Dentistry | 4 | 4 |
| Dermatology | 3 | 8 |
| Diabetes | 7 | 2 |
| Emergency Medicine | 2 | 12 |
| Endocrinology | 5 | 3 |
| Epidemiology | - | 2 |
| ENT | 6 | 2 |
| Family Medicine | 6 | 13 |
| Gastroenterology | 18 | 6 |
| General Surgery | 3 | 3 |
| Geriatrics | - | 1 |
| Hematology | - | 1 |
| Infectious Diseases | 4 | 7 |
| Intensive Care | 6 | 1 |
| Internal Medicine | 29 | 17 |
| Laboratory Medicine | 1 | 4 |
| Neonatology | 1 | 4 |
| Nephrology | 1 | 5 |
| Neurology | 7 | 4 |
| Neuropediatrics | - | 1 |
| Neurosurgery | 3 | - |
| Gynecology | 7 | 2 |
| Occupational Medicine | 2 | - |
| Oncology | 3 | - |
| Ophthalmology | 8 | 4 |
| Orthopedics | 3 | 2 |
| Other | 10 | 3 |
| Pathology | - | 2 |
| Pediatrics | 1 | 11 |
| Physical Medicine | - | 2 |
| Pneumology | 2 | 7 |
| Preventive Medicine | - | 1 |
| Psychiatry | 2 | 6 |
| Radiology | 8 | 11 |
| Radiation Oncology | - | 1 |
| Rheumatology | 27 | 18 |
| Urology | 1 | 1 |
